# Spinal myeloid sarcoma presenting as initial symptom in acute promyelocytic leukemia with a rare cryptic *PLZF::RARα* fusion gene: a case report and literature review

**DOI:** 10.3389/fonc.2024.1375737

**Published:** 2024-05-21

**Authors:** Xuejiao Zhang, Tao Wang, Pu Chen, Yan Chen, Zhimei Wang, Tianhong Xu, Pengfei Yu, Peng Liu

**Affiliations:** ^1^ Department of Hematology, Zhongshan Hospital, Fudan University, Shanghai, China; ^2^ Department of Hematology, Zhongshan Hospital (Minhang Meilong Branch), Fudan University and Shanghai Geriatric Medical Center, Shanghai, China; ^3^ Key Laboratory of Digital Technology in Medical Diagnostics of Zhejiang Province, Dian Diagnostics Group Co. Ltd., Hangzhou, Zhejiang, China; ^4^ Department of Laboratory Medicine, Zhongshan Hospital, Fudan University, Shanghai, China

**Keywords:** spine, myeloid sarcoma, acute promyelocytic leukemia, *PLZF::RARα* fusion, cryptic

## Abstract

**Background:**

Acute promyelocytic leukemia (APL) is rarely caused by the *PLZF::RARα* fusion gene. While APL patients with *PLZF::RARα* fusion commonly exhibit diverse hematologic symptoms, the presentation of myeloid sarcoma (MS) as an initial manifestation is infrequent.

**Case presentation:**

A 61-year-old patient was referred to our hospital with 6-month history of low back pain and difficulty walking. Before this admission, spine magnetic resonance imaging (MRI) conducted at another hospital revealed multiple abnormal signals in the left iliac bone and vertebral bodies spanning the thoracic (T11-T12), lumbar (L1-L4), and sacral (S1/S3) regions. This led to a provisional diagnosis of bone tumors with an unknown cause. On admission, complete blood count (CBC) test and peripheral blood smear revealed a slightly increased counts of monocytes. Immunohistochemical staining of both spinal and bone marrow (BM) biopsy revealed positive expression for CD117, myeloperoxidase (MPO), and lysozyme. BM aspirate showed a significant elevation in the percentage of promyelocytes (21%), which were morphologically characterized by round nuclei and hypergranular cytoplasm. Multiparameter flow cytometry of BM aspirate revealed that blasts were positive for CD13, CD33, CD117, and MPO. Through the integrated application of chromosome analysis, fluorescence *in situ* hybridization (FISH), reverse transcriptase polymerase chain reaction (RT-PCR), and Sanger sequencing, it was determined that the patient possessed a normal karyotype and a rare cryptic *PLZF::RARα* fusion gene, confirming the diagnosis of APL.

**Conclusion:**

In the present study, we report the clinical features and outcome of a rare APL patient characterized by a cryptic *PLZF::RARα* fusion and spinal myeloid sarcoma (MS) as the initial presenting symptom. Our study not only offers valuable insights into the heterogeneity of APL clinical manifestations but also emphasizes the crucial need to promptly consider the potential link between APL and MS for ensuring a timely diagnosis and personalized treatments.

## Introduction

Acute promyelocytic leukemia (APL), also known as acute myeloid leukemia (AML) subtype M3 according to the French-American-British (FAB) classification, is primarily characterized by an accumulation of immature promyelocytes in bone marrow (BM) (1). APL patients typically appear as one or more of hematologic symptoms, including fever, bleeding, fatigue, infections, bone pain, and others (1). Besides, in some cases, APL may present with extramedullary involvement that causes myeloid sarcoma (MS) (2–6). Although rare in clinical practice, MS is more commonly associated with relapsed or refractory APL cases, with an estimated incidence of 3%–5% (2). However, in newly diagnosed APL, MS occurs even more rarely, potentially contributing to delays in APL diagnosis (3–6). In addition, MS can occur simultaneously in various extramedullary locations, such as skin, soft tissues, bones, lymph nodes, and other organs (3–6). Therefore, MS may produce miscellaneous non-hematologic symptoms that mimic those of other diseases, making it more challenging to timely distinguish MS. Moreover, the atypical morphological characteristics exhibited by leukemia cells at onset of this disease adds complexity to the diagnostic procedure in cases of APL with MS (4–6). Given the substantial risk of disseminated intravascular coagulation (DIC) in association with APL, a condition that can be severe and life-threatening, it is imperative to prioritize early APL diagnosis and the immediate commencement of APL-specific treatments like all-trans retinoic acid (ATRA) and arsenic trioxide (ATO) (1, 7, 8).

One of the key diagnostic features of APL is chromosomal translocation involving the gene that encodes retinoic acid receptor alpha (RARα) on chromosome 17 (1, 9–11). In particular, an overwhelming majority of APL cases exhibit the typical t(15;17)(q22;21) translocation, which results in the fusion of the promyelocytic leukemia (*PML*) gene and *RARα* gene, namely *PML::RARα* fusion gene (9). In exceptionally infrequent cases (1~2%), APL has been observed with rare variant translocations, including t(11;17)(q23;q21), t(11;17)(q13;q21), t(5;17)(q32;q21), and t(17;17)(q11;q21) (10, 11). These variant translocations involve other partner genes and may impact on the clinicopathologic features of APL. For example, APL patients with the classic *PML::RARα* fusion gene are highly responsive to ATRA and ATO (1, 9). However, patients with the t(11;17)(q23;q21) translocation, resulting in the fusion of the promyelocytic leukemia zinc finger (*PLZF*)-encoding gene and *RARα* gene—a fusion less prevalent than *PML::RARα*—may display a comparatively less robust response to the same treatments (10–12). It is important to note that some thirty APL cases with MS as initial presentation has been documented in literatures so far, and almost all carried the classic t(15;17)(q22;21) translocation (3–6). The development of MS is still scarcely reported in APL with other rare variant translocations. Here, we report a newly diagnosed APL patient (61-year-old male) with spinal MS as the first presentation. Using integrated genetic testing, we identified a normal karyotype, and notably, a rare cryptic *PLZF::RARα* fusion gene.

## Case presentation

A 61-year-old Chinese man was referred to our hospital with 6-month history of low back pain and difficulty walking, which were particularly severe after physical exertion. The patient reported occasional temporary relief of these symptoms through Chinese medical massage treatments. One month before being admitted, his symptoms had worsened progressively without a clear precipitating factor, and he experienced pain that extended to his left thigh accompanied by a sensation of numbness. Subsequently, detailed bone examinations were performed at local hospital. At that time, spine magnetic resonance imaging (MRI) suggested pathologic fracture of lumbar (L3) spine and showed multiple abnormal signals in left iliac bone and in vertebral bodies of the thoracic (T11-T12), lumbar (L1-L4), and sacral (S1/S3) spine. Meanwhile, fluorine-18-fluorodeoxyglucose (FDG) positron emission tomography/computed tomography (PET/CT) imaging reported that FDG uptake was slightly increased in those bone lesions, but not in other areas of the body. The patient was tentatively diagnosed with bone tumors of unknown cause and was transferred to our hospital for further diagnosis and treatment.

**Figure 1:**
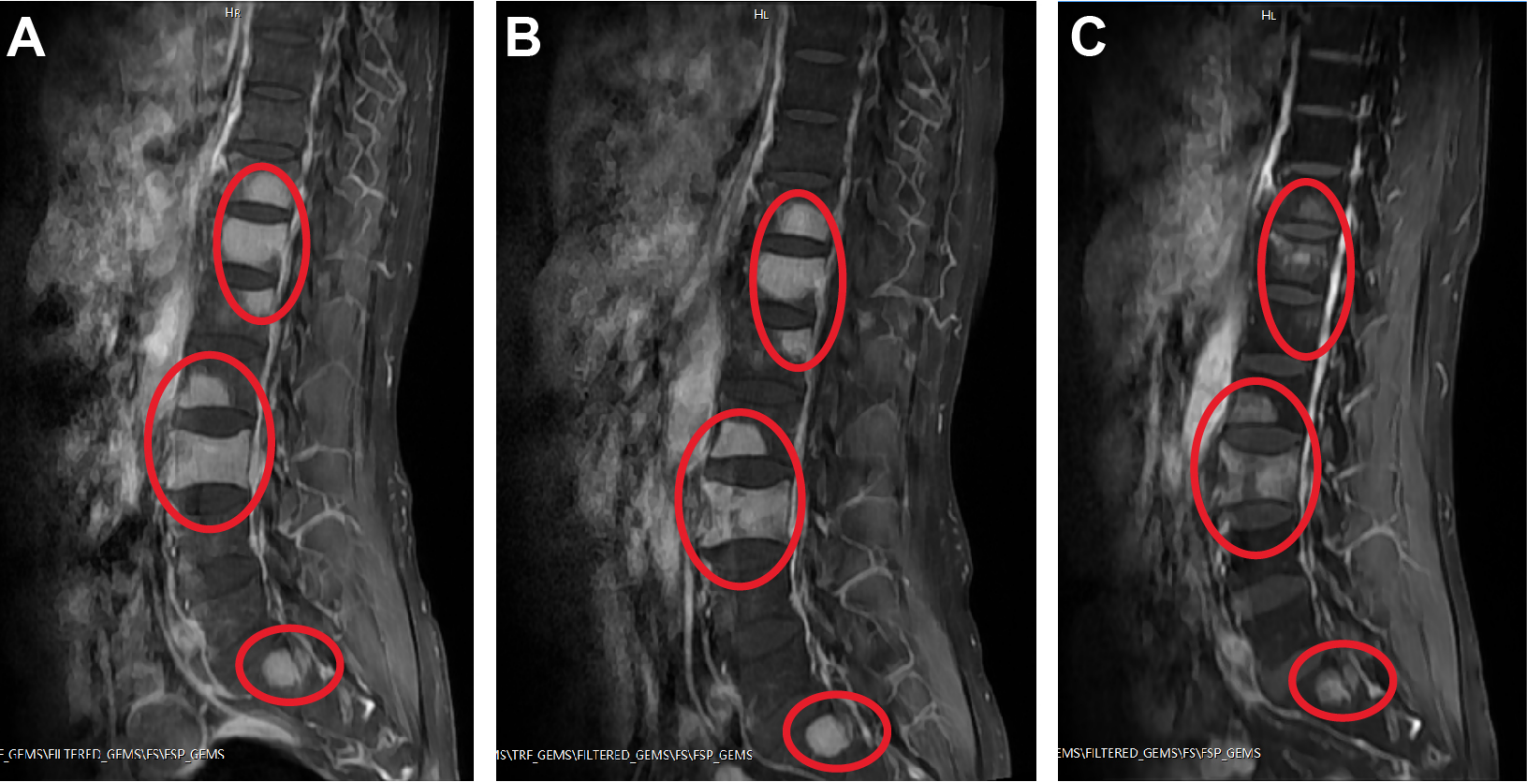
Examples of spinal sagittal T1-weighted MRI of the patient. **(A)** On admission. **(B)** After first cycle of therapy. **(C)** After second cycle of therapy. Abnormal high signals on T1 images are shown circled in red.

On admission, our spine MRI confirmed the previous results (**Figure 1A**). A complete blood count (CBC) test showed a slightly increased counts of monocytes (0.73×10^9^/L; normal range: 0.1-0.6×10^9^/L), and normal results of red blood cells (4.95×10^12^/L), hemoglobin (142 g/L), platelets (204×10^9^/L), and white blood cells (7.46×10^9^/L) (**Table 1**). Examination of the peripheral blood smear consistently suggested an increased proportion of monocytes. Moreover, antibody serology tests revealed positive results for antinuclear (ANA) antibody and anti-beta-2 glycoprotein 1 (B2GP1) antibody. With flow cytometry (Becton Dickinson FastImmune™ Cytokine System), we detected elevated levels of interferon gamma (IFN-γ; 13.03 pg/mL; normal range:≤4.43 pg/mL) and interleukin-17A (5.60 pg/mL; normal range:≤4.74 pg/mL) in whole blood. Notably, coagulation tests revealed a significant increase in D-dimers level (4.12 mg/L; normal range: 0-0.8 mg/L), while other coagulation indexes were within normal range (**Table 1**). Heart, renal, liver, and nervous system functions were normal.

**Table 1:** The patient’s blood cells counts and conglation indexs at different stages.

	1st cycle of therapy		2nd cycle of therapy		normal range
	before	after	before	after	
**red blood cells (×10^12^/L)**	4.95	4.94	4.87	3.12	4.30-5.80
**hemoglobin (g/L)**	142	145	143	92	130-175
**platelets (×10^9^/L)**	204	221	268	77	125-350
**white blood cells (×10^9^/L)**	7.46	3.9	2.8	1.8	3.50-9.50
**PT (s)**	11.3	10.9	12	11.8	10.0-13.0
**INR**	0.97	0.94	1.04	1.02	0.5-1.2
**TT (s)**	15.7	15.8	15	15.9	14.0-21.0
**aPTT (s)**	29.5	28	27.9	26.5	25.0-31.3
**fibrinogen (mg/dL)**	252	270	211	316	200-400
**D-dimer (mg/L)**	4.12	1.21	0.76	0.39	0-0.8
**FDP (μg/mL)**	6.78	2.5	2.5	2.5	<10

PT, prothrombin time; INR, the international normalized ratio; TT, thrombin time; aPTT, activated partial thromboplastin time; FDP, fibrin degradation products.

**Figure 2:**
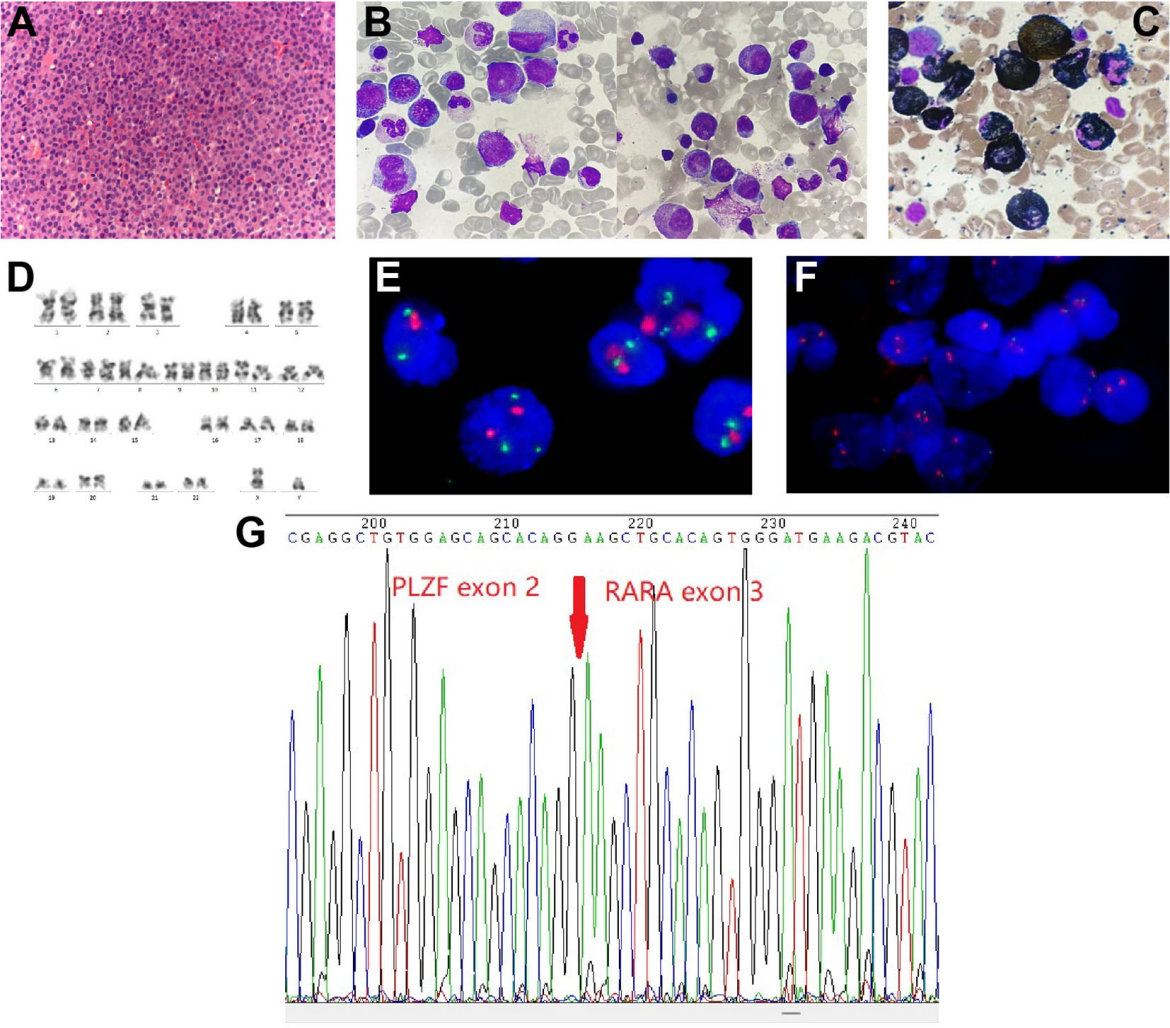
Spine tissue and bone marrow (BM) aspirate examinations and results of karyotype and fluorescence *in situ* hybridization (FISH) analysis. **(A)** Histopathology of spinal cord (hematoxylin and eosin staining; magnification, ×400). **(B)** BM aspirate shows atypical acute promyelocytic leukemia morphology (Wright-Giemsa staining; magnification, ×1,000). **(C)** BM aspirate showed that promyelocytes were myeloperoxidase (MPO) positivity (MPO staining; magnification, ×1,000). **(D)** Chromosome analysis of the BM reveals a normal male karyotype. **(E, F)** Metaphase FISH analysis of the patient. **(E)** The standard dual-color, dual-fusion probe set probe set for t(15;17) shows the presence of two red signals (*PML*) and three green signals (*RARα*), suggesting *RARα* rearrangement; **(F)** The *RARα* break-apart probe detects separated red signals (*RARα*). **(G)** Sanger sequencing analysis demonstrates *PLZF*::*RARα* fusion transcript.

In order to assess the histopathological basis of bone lesions, we further performed CT-guided percutaneous needle biopsy of lumbar L3. The spinal biopsy results indicated the presence of immature/blast-like cells with eccentric nuclei within the spaces of the bone trabeculae (**Figure 2A**). Immunohistochemical staining of the spinal biopsy revealed positive expression for CD117, CD43, myeloperoxidase (MPO), lysozyme, and Ki67 (labelling index about 40%), while it tested negative for CD20, CD34, CD56, CD61, CD79a, CD138, and IgG/M, κ, λ expression. These findings suggested the presence of myeloid neoplasms. Meanwhile, BM biopsy revealed 95% of blast cells and a staining profile characterized by CD117 (+), MPO (+) and lysozyme (part+), which was similar to the results of spinal biopsy. In addition, BM aspirate showed hypercellularity with an elevated myeloid/erythroid (M/E) ratio of 7.52:1. Specifically, there was a significant elevation in the percentage of promyelocytes (21%; normal range: 0.4-3.9%), strongly indicating the likelihood of APL. Erythropoiesis was insufficient, while megakaryopoiesis was normal. Giemsa-stained promyelocytes displayed round nuclei and hypergranular cytoplasm (**Figure 2B**). However, Auer rods were notably absent. The majority of promyelocytes had positive staining for MPO (**Figure 2C**). Multiparameter flow cytometry of BM aspirate detected 78% blasts and suggested an immunophenotype that was positive for CD13, CD33, CD117, and MPO, and negative for CD3, CD10, CD11b, CD14, CD15, CD19, CD34, CD71, CD79a, and HLA-DR, corresponding to APL features.

Notably, cytogenetics G-band analysis of BM cells revealed a normal male karyotype (46, XY) (**Figure 2D**). Metaphase fluorescence *in situ* hybridization (FISH) analysis with the *PML::RARα* dual-color dual-fusion probe kit (FP-005, Wuhan HealthCare Biotechnology Co., Ltd.) on BM aspirate suggested the absence of *PML*-*RARα* dual-fusion translocation (**Figure 2E**). However, three green FISH signals suggested the presence of *RARα* translocation (**Figure 2E**). This finding was subsequently validated using the *RARα* break-apart probe detection kit (FP-043, Wuhan HealthCare Biotechnology Co., Ltd.) (**Figure 2F**). To further explore the etiology, we performed reverse transcriptase polymerase chain reaction (RT-PCR) (Dian Diagnostics Group Co. Ltd., Hangzhou, China) on BM. It revealed *PLZF::RARα* fusion by using the reverse primers (NM_000964; *RARα* 1-R, 5’-AAGCCCTTGCAGCCCTCAC-3’ [external]; *RARα* 2-R, 5’-CCCATAGTGGTAGCCTGAGGAC-3’ [internal]) located within exon 2 of *RARα* gene in conjunction with the forward primers (NM_001018011; *PLZF* 1-F, 5’-CCACAAGGCTGACGCTGTATT-3’ [external]; *PLZF* 2-F, 5’-GTGGGCATGAAGTCAGAGAGC-3’ [internal]) located within exon 3 of *PLZF* gene. Sanger sequencing further confirmed the presence of *PLZF::RARα* exon 3–exon 2 fusion transcript (**Figure 2G**). Next-generation sequencing (NGS) analysis with the Myeloid Tumor Assay that was consisted of 128 genes panel (Dian Diagnostics Group Co., Hangzhou, China) detected no additional mutations. Taken together, according to FAB classification, a definitive diagnosis of APL was ultimately established.

In the initial induction therapy, the patient was treated with 20 mg/day ATRA (BID) for one week. This was followed by a regimen incorporating subcutaneous azacitidine (120 mg/day, Day 1 to 7) and oral administration of venetoclax with a progressive dose escalation: 100 mg/day (Day 1), 200 mg/day (Day 2), and 400 mg/day (Day 3 to 24) (**Table 2**). During this period, the patient was also treated with cetirizine for skin itch and rash. Subsequent CBC revealed that his WBC counts reduced to 3.9×10^9^/L, which was still within normal range (**Table 1**). BM aspirate showed hypercellularity and a decreased M/E ratio of 0.2:1, which was characterized by granulocytic hypoplasia and erythrocytic/megakaryocytic hyperplasia. Importantly, BM aspirate indicated that the percentage of promyelocytes reduced to 0.5%. On BM biopsy, residual leukemia cells were negligible. However, spine MRI showed no significant improvement in MS lesions (**Figure 1B**). RT-PCR from BM showed the persistence of *PLZF::RARα* fusion.

**Table 2:** Main clinical characteristics and treatment of the patient.

	Clinical characteristics				Treatment regimen(s)
	spinal MS	BM APL	other syptoms	*PLZF::RARα* fusion	
1st cycle of therapy	Yes	Yes	skin itch, rash	Yes	1. ATRA; 2. venetoclax and azacitidine; 3. cetirizine.
2nd cycle of therapy	Yes	No	pancytopenia, agranulocytosis	Yes	1. venetoclax and idarubicin; 2. herombopag, IL-11, and blood transfusion.
after 2nd cycle of therapy	Yes, but in remission	No	/	No	/

APL, acute promyelocytic leukemia; MS, myeloid sarcoma; BM, bone marrow; ATRA, all-trans retinoic acid; IL-11, interleukin-11.

As a result, we maintained the patient on oral venetoclax administration at 400 mg/day (Day 1 to 12) and further administered idarubicin intravenously (10 mg/day IV bolus, Day 1 and 2; 20 mg/day IV bolus, Day 3) (**Table 2**). Meanwhile, the patient developed pancytopenia, and had sustained agranulocytosis for two weeks. To address this, herombopag, recombinant human interleukin-11 (IL-11), and blood transfusion were given (**Table 2**). Repeated BM aspirate showed reduced cellularity and a decrease in all three blood cell lineages. Notably, the percentage of promyelocytes increased again to 12%, but subsequent flow cytometry immunophenotyping confirmed a normal phenotype of immature granulocytes, which was hypothesized to be a possible manifestation of myeloid hematopoietic recovery. Fortunately, MRI showed that spinal MS lesions were obviously shrunken (**Figure 1C**). The patient also obtained symptomatic relief of low back pain and difficulty walking. What’s more important, *PLZF::RARα* fusion transcript became undetectable, indicating the achievement of complete molecular remission (MR). The decision to initiate additional treatment was contingent upon the successful recovery of the patient’s hematopoietic functions.

## Discussion

According to the new International Consensus Classification (ICC) of myeloid neoplasms and acute leukemias, APL with t(11;17)(q23;q21) translocation is now redefined as APL with other *RARα* rearrangements (13). Since the first report in 1993, only about forty newly diagnosed APL patients with t(11;17)(q23;q21) have been documented in literatures (**Table 3**). This rare APL impacts individuals across a broad spectrum of ages, ranging from 15 to 81 years old, with an average age of 48.8 years (**Table 3**). Interestingly, the prevalence of APL with t(11;17)(q23;q21) appears to be higher in males (35/41; 85.4%) compared to females (6/41; 14.6%) (**Table 3**). The t(11;17)(q23;q21) translocation gives rise to *PLZF::RARα* fusion gene, also referred to as *ZBTB16::RARα*. *PLZF* exhibits the ability to bind to DNA, thereby governing the transcriptional activity of genes pivotal to diverse cellular functions, particularly those involved in the differentiation and maturation of promyelocytic cells (40). However, it’s important to highlight that, in very rare APL cases, the karyotype may appear normal, and the fusion gene may be formed through cryptic or subtle rearrangements that are not readily detected by standard cytogenetic analysis (19, 41). Similar to our patient, Grimwade D et al. previously reported an APL case with a normal karyotype and cryptic formation of the *PLZF::RARα* fusion gene (19). Meanwhile, studies suggested that cryptic formation was not only limited to *PLZF::RARα*, but also identified in APL with *PML::RARα* (41, 42)*, IRF2BP2::RARα* (43)*, TBL1XR1::RARα* (44), and *FIP1L1::RARα* (45). Such exceptional APL cases underscore the critical importance of employing molecular techniques, such as FISH or RT-PCR, to pinpoint the precise genetic abnormality and confirm the final diagnosis of APL.

**Table 3:** APL patients harboring *PLZF::RARα* reported in the literature.

Patient	Age/Sex	Karyotypic anomaly	Initial symptom(s)	WBC (^10^9^/L)	Auer rods	Treatment regimen(s)	HSCT	Prognisis	Reference
1	67/male	46,XY,t(11;17)(q23;21)	weakness, anorexia, coughing, gingival bleeding	4.1	No	ATRA	No	Died of pneumonia and respiratory failure (at day 20)	Chen SJ, et al. (12)
2	68/male	t(11;17)(q23.24;12.21)	NA	10.6	Yes	1. Daun and Ara-C; 2. ATRA; 3. Ara-C and MTZ.	No	Died of septic shock (at month 11)	Guidez F, et al. (14)
3	81/male	46,XY,t(11;17)(q23;21)	fatigue, dyspnea, fever, and bone pain	7.6	Yes	ATRA	No	Died of brain stem hemorrhage (at day 18)	Licht JD, et al. (15)
4	37/female	46,XX,t(11;17)(q23;21)	NA	45.2	NA	1. Daun and Ara-C; 2. α-interferon; 3. MTZ and ETO.	No	Died of unknown reason (at month 11)	
5	34/male	46,XY,t(11;17)(q23;21)	bone pain and neutropenia	2.4	Yes	1. ATRA; 2. Daun and Ara-C; 3. amsacrine and Ara-C.	No	CR (4 months)	
6	53/male	46,XY,+(3)+(13)(q34), t(11;17)(q23;q21)	spontaneous bruising	15.3	NA	1. Daun and Ara-C; 2. Daun, Ara-C, and G-CSF; 3. MTZ and ETO; 4. prednisone, vincristine, 6-MP, and MTZ; 5. ATRA; 6. IDA; 7. fludarabine and Ara-C.	No	Died of congestive heart failure, DIC, and acute renal failure (at month 31)	
7	53/male	46,XY,t(11;17)(q23;q12-21)	fatigue, shortness of breath, spontaneous bruising	4.5	No	1. ATRA, Ara-C, Daun, ETO, and G-CSF; 2. Ara-C, Daun, and ETO; 3. amsacrine, Ara-C, and ETO; 5. MTZ and Ara-C.	No	CR (10 months)	Culligan DJ, et al. (16)
8	41/male	t(11;17)(23;q21)	schizophrenia, blasts in peripheral blood	7.8	Yes	1. ATRA, Ara-C, Daun, 6-MP, and PDN; 2. MTZ, Ara-C, 6-MP, and PDN.	No	Died of intracranial invasion and DIC	Hoshi S. Rinsho Ketsueki (17).
9	31/male	t(11;17)(23;q21)	hyperleukocytosis	69	Yes	1. ATRA; 2.Daun and Ara-C; 2. Ara-C and amsacrine; 3. MTZ and ETO; 4. ATRA and G-CSF.	allo-HSCT	CR (51 months)	Jansen JH, et al. (18)
10	32/male	45,X,-Y,t(11;17)(q23;q21)	NA	11.6	NA	1. ATRA; 2. Daun and Ara-C.	allo-HSCT	CR (37 months)	Grimwade D, et al. (19)
11	43/male	46,XY,i(7)(q10),t(11;17)(q23;q21)	NA	10.4	NA	ATRA and IDA	auto-HSCT	Died of APL replase (at month 30)	
12	34/male	46,XY,del(11)(q23)/45,idem,-Y/46,XY	NA	20	NA	1. Daun, Ara-C, and ETO; 2. Ara-C, IDA, and ATRA.	allo-HSCT	CR (33 months)	
13	62/male	46,XY.ish,ins(11;17)(q23;q21,q21)	NA	9.9	NA	1. Ara-C, IDA, and ETO; 2. Ara-C, ETO, gemtuzumab ozogamicin, IDA, MTZ.	No	CR (15 months)	
14	75/male	46,XY,t(11;17)(q23;q21)/46,idem,del(12)(p1)?/46,idem,-6,+r	NA	2	NA	1. ATRA, Daun, Ara-C, and thioguanine; 2. amsacrine, Ara-C, and ETO.	No	CR (17 months)	
15	23/male	46,XY,t(11;17)(q23;21)	fever, bone pain (left hip and shoulder)	9.1	NA	1. ATO; 2. Ara-C and Daun; 3. Ara-C; 4. Ara-C and Daun.	No	CR (32 months)	George B, et al. (20)
16	83/male	46,XY,t(11;17)(q23;21)	NA	NA	NA	1. ATRA and Daun; 2. Daun and Ara-C; 3. ATRA, 6-MP, and MTZ.	No	CR (24 months)	Cassinat, B, et al. (21)
17	50/male	46,XY,t(11;17)(q23;q21)/45,X,-Y,t(11;17)(q23;q21)	NA	6.8	NA	1. ATRA, Ara-C, Daun, and ETO; 2. Ara-C, Daun, and ETO; 3. amsacrine, Ara-C, and ETO; 4. MTZ and Ara-C.	No	CR (73 months)	Jovanovic JV, et al. (22)
18	52/male	46,XY,t(11;17)(q23;21)	pancytopenia	1.62	Yes	chemotherapy without ATRA	No	NA	Han SB, et al. (23)
19	38/female	46,XX,t(11;17)(q23;21)	fever, dyspnea, chest pain	23.6	Yes	1. ATRA, Daun, and Ara-C; 2. MTZ, ETO, and Ara-C.	No	Died of sepsis with active disease	Rohr SS, et al. (24)
20	48/male	46,XX,t(11;17)(q23;21)	weight loss, fatigue, tonsillitis	71.6	Yes	1. Daun and ATRA; 2. Daun and Ara-C; 3. Ara-C and ATO.	allo-HSCT	PR	
21	60/female	46,XX,der (11),der(17)	fever, dizziness, fatigue	34	NA	1. ATRA and hydroxyurea; 2. ATRA, MTZ, Ara-C, and ATO; 3. ATO and chemotherapy.	No	CR (11 months)	Liu KQ, et al. (25)
22	23/male	t(11;17)(q23;q21)	fever, shortness of breath, leg swelling	NA	Yes, but few	Refuse chemotherapy	No	NA	Palta A, et al. (26)
23	49/female	46,XX,del(5)(q13q31),t(11;17)(q23;q21)	rheumatoid arthritis, pancytopenia	7.9	No	1. ATRA, IDA, Ara-C, and ETO; 2. ATRA, Ara-C, and MTZ,	allo-HSCT	CR	Piñán MA, et al. (27)
24	50/male	t(11;17)(q23;q21)	fever, knee pain	NA	No	1. Ara-C and Daun; 2. ATRA and ATO.	No	CR	Lechevalier N, et al. (28)
25	53/male	46,XY, t(11;17)(q23;q21) with del(5)(q22q35)	Crohn disease and macrocytic anemia	15.4	No	1. ATRA; 2. Daun and Ara-C	No	NA	Dowse RT, et al. (29)
26	46/male	46,XX,t(11;17)(q23;21)	fever, leg swelling	35.5	NA	1. ATRA and ATO; 2. Ara-C and IDA; 3. MTZ, ETO, and Ara-C; 4. MTZ, Ara-C; 5. Ara-C; 6. pirarubicin and Ara-C	No	CR	Wen HX, et al. (30)
27	81/female	46,XX,add(17)(q21) (4)/46,XX (9). ish der(11)t(11;17)(q23;q21)	back pain	NA	NA	ATRA	No	Died of pulmonary hemorrhage (at day 10)	Langabeer SE, et al. (31)
28	48/female	46,XX,t(11;17)(q23;q21);47,idem,+22	NA	42.5	NA	ATRA, hydroxycarbamide	No	Died of cerebral bleeding (at 0.3 months)	Wang XX, et al. (32)
29	44/male	46,XY,t(11;17)(q23;21)	bone pain (lower limbs and hip)	52.07	NA	ATO, Daun, and Ara-C	No	NR	
30	52/male	47,XY,+8/47,idem, t(11,17)(q23,q21)	fever, gingival bleeding	8.92	NA	1. ATRA and ATO; 2. Daun and Ara-C; 3. ATRA, Ara-C, aclarubicin, and G-CSF.	No	CR (7 months)	
31	62/male	46,XY,t(11;17)(q23;21)	gout, pancytopenia	2.99	Yes, but few	1. Ara-C and IDA; 2. Ara-C, IDA, and ATRA; 3. Ara-C and ATRA	No	CR	Pardo Gambarte L, et al. (33)
32	51/male	46,XY,t(11;17)(q23;q21) [18]/47,idem,+8 [2]	fatigue, easy bruising	NA	NA	NA	No	NA	Liu G, et al. (34)
33	56/male	t(11;17)(q23;q21)	apnoea, night sweats	25.47	No	IDA and Ara-C	allo-HSCT	CR (2 years)	Canali A and Rieu JB (35).
34	44/male	45,X,-Y,t(11;17)(q23;q21)	flu-like illness	NA	No	fludarabine, Ara-C, G-CSF, IDA, and venetoclax.	No	CR	Courville EL, et al. (36)
35	56/male	46,XY,add (9)(q11)	lower extremity paralysis	7.1	No	NA	NA	NA	Cho EJ, et al. (37)
36	66/male	t(11;17)(q23;q21)	fever, weight loss, arthralgia	11.1	No	1. steroids; 2. Ara-C and Daun	allo-HSCT	CR (1.5 years)	Castelijn DAR, et al. (38)
37	15/male	NA	abdominal pain, weakness, fever	64.94	No	1. IDA and ATO; 2. Ara-C; 3. ATRA	No	CR	Rabade N, et al. (39)
38	38/male	NA	easy fatigability, dyspnea, and fever	NA	Yes	ATO	No	Died of unknown reseaon (at 2 months)	
39	45/male	NA	easy fatigability and fever	NA	No	ATO	No	NA	
40	36/male	NA	fever and rash	4.86	No	1. decitabine and ATO; 2. Daun and Ara-C; 3. Ara-C	No	Death in relapse (at 11 months)	
41	22/male	NA	fever and body ache	76.99	No	1. ATO, Daun, and Ara-C; 2. Ara-C	No	NA	

WBC, white blood cells; NA, not available; ATRA, all-trans-retinoic acid; ATO, arsenic trioxide; Daun, daunorubicine; Ara-C, cytarabine; MTZ, mitoxantrone; ETO, etoposide; G-CSF, granulocyte colony-stimulating factor; 6-MP, 6-mercaptopurinum; IDA, idarubicin; PDN, prednisolone; CR, complete remission; PR, partial remission; NR, no response; HSCT, hematopoietic stem cell transplantation; allo-HSCT, allogeneic hematopoietic stem cell transplantation; auto-HSCT, autologous hematopoietic stem cell transplantation.

Moreover, it’s noteworthy that a majority of APL patients harboring the *PLZF::RARα* fusion initially manifest with non-specific symptoms that were identical to classical APL, including fever, pancytopenia, fatigue, bone pain, and so forth (**Table 3**). MS is generally considered a rare extramedullary manifestation of untreated APL, but after induction therapy MS becomes more common (2, 3). As of our current information, our patient was actually the second report of APL with *PLZF::RARα* fusion and MS as the initial symptom. The previous case was a 56-year-old Korean man characterized by APL and spinal MS (37). Even in classical APL, only around thirty cases with MS have been reported thus far (3–6). In addition, a recent report by Wang, Y., et al. highlighted the presence of skull MS in a 28-month-old girl with APL caused by *FIP1L1::RARα* fusion (45). The fact that MS has been identified in APL with different variants suggests that MS may not be exclusive to a particular genetic fusion. However, the exact mechanism underlying the development of MS in APL is not fully understood, and it may involve various processes related to the behavior of leukemia cells. In patients with AML or APL, MS can manifest in various sites throughout the body. Bone represents a frequent site of involvement, with MS lesions often observed in spine, skull and long bones (4, 6, 45–47). Additionally, soft tissues including skin, subcutaneous tissue, and lymph nodes are susceptible to MS infiltration (2, 6, 48). In more severe cases, MS can affect visceral organs such as liver, colon, and central nervous system (CNS) (5, 49, 50). The presentation of MS varies widely based on the affected site(s), necessitating a comprehensive diagnostic approach and tailored treatment strategies.

Morphological characteristics of abnormal promyelocytes exhibit variability among APL patients with the *PLZF::RARα* fusion, occasionally differing significantly from those seen in classic APL (11, 29, 33, 51). In classic APL, distinguishing features of promyelocytes encompass lobulated nuclei, hypergranular cytoplasm, and Auer rods (1, 51). However, a subgroup of APL patients with the *PLZF::RARα* fusion, similar to our patient, may present with atypical traits, such as round/non-lobulated nuclei, hypogranular or entirely agranular cytoplasm, along with the absence of Auer rods (**Table 3**). Notably, studies have found that APL cases with the *PLZF::RARα* fusion may exhibit vacuoles or square crystalline structures within the cytoplasm of promyelocytes (29, 33). Interestingly, we also observed small vacuoles in few abnormal promyelocytes from our patient. Further research is needed to better understand the underlying mechanisms leading to the formation of these atypical intracytoplasmic inclusions and their clinical significance. Hence, in instances with atypical presentations, the use of stains like MPO, Sudan Black B, and immunohistochemical markers such as CD13, CD33, and CD117 can be valuable in reinforcing the diagnosis of APL (1, 13). Nevertheless, it should be noted that APL patients may infrequently show negativity for both MPO and Sudan Black B staining (52, 53), and the immunophenotype may also undergo changes after induction therapy (54).

The immediate initiation of ATRA is now a crucial element in the induction therapy for classic APL, resulting in a notable rise in complete remission (CR) rates and enhanced overall outcomes (8, 9). Currently, there is no established consensus guideline regarding the utilization of ATRA in the treatment of APL with rare variants and MS. Despite demonstrating the ability of leukemia cells carrying the *PLZF::RARα* fusion to fully differentiate with both *ex vivo* and *in vivo* ATRA treatment, the clinical reality is that APL with this rare fusion is commonly considered ATRA-insensitive and is linked to an unfavorable prognosis (10–12, 55). Significantly, it’s also been reported that a small number of APL patients with *PLZF::RARα* fusion who underwent a combination of ATRA and intensive chemotherapy achieved CR (11, 33). In recent years, the BCL-2 inhibitor venetoclax has exhibited encouraging therapeutic outcomes in AML as well as other hematological malignancies (56). Interestingly, exploratory studies suggested that APL patients who are resistant to conventional chemotherapies may derive benefit from regimens incorporating venetoclax (57). In particular, APL patients harboring exceedingly uncommon *RARα::HNRNPC* and *RARα::THRAP3* fusions have been documented to achieve CR through the administration of venetoclax and hypomethylating agents such as azacytidine or decitabine (58, 59). These findings prompted us to initiate treatment with ATRA, followed by a combination of venetoclax and azacytidine in our patient. The treatment demonstrated a significant efficacy in eradicating leukemic cells from BM aspirate; however, its impact on alleviating his MS and achieving MR was negligible. Fortunately, the substitution of azacitidine with the anthracycline antineoplastic agent idarubicin has ultimately led to the achievement of MR, albeit the occurrence of significant hematological toxicity.

## Conclusions

To summarize, we report the clinical features and outcome of a rare APL patient characterized by a cryptic *PLZF::RARα* fusion and MS as the initial presenting symptom. Our study not only offers valuable insights into the heterogeneity of APL clinical manifestations but also emphasizes the crucial need to promptly consider the potential link between APL and MS for ensuring a timely diagnosis and personalized treatments.

## Data availability statement

The raw data supporting the conclusions of this article will be made available by the authors, without undue reservation.

## Ethics statement

The studies involving humans were approved by the ethics committee of Zhongshan Hospital of Fudan University. The studies were conducted in accordance with the local legislation and institutional requirements. The participants provided their written informed consent to participate in this study. Written informed consent was obtained from the individual(s) for the publication of any potentially identifiable images or data included in this article.

## Author contributions

XZ: Data curation, Investigation, Writing – original draft. TW: Formal analysis, Investigation, Writing – review & editing. PC: Formal analysis, Writing – review & editing. YC: Formal analysis, Writing – review & editing. ZW: Project administration, Writing – review & editing. TX: Investigation, Writing – review & editing. PY: Investigation, Writing – review & editing. PL: Funding acquisition, Supervision, Writing – review & editing.
